# Successful Cardiac Resynchronization Therapy Defibrillator Implantation via the Vein of Marshall in a Patient with Coronary Sinus Ostial Occlusion

**DOI:** 10.19102/icrm.2025.16094

**Published:** 2025-09-15

**Authors:** Farid Aliyev, Emin Karimli, Aytan Hajili

**Affiliations:** 1 Department of Cardiology, Baku Health Center, Baku, Azerbaijan; 2 Department of Cardiology, Universal Hospital, Baku, Azerbaijan

**Keywords:** Coronary sinus, cardiac resynchronization therapy, heart failure

## Abstract

Cardiac resynchronization therapy (CRT) is a well-established treatment for patients with heart failure and wide QRS complexes. Successful left ventricular (LV) lead implantation is typically achieved through the coronary sinus (CS). However, congenital anomalies such as CS ostial atresia can complicate the procedure. We report a case of a 65-year-old man with a history of aortic valve replacement and heart failure who underwent successful CRT-defibrillator implantation. During the procedure, CS ostial atresia was unexpectedly encountered, preventing standard venous access. The LV lead was successfully implanted via the vein of Marshall.

## Background

Cardiac resynchronization therapy (CRT) has become a cornerstone in the management of patients with heart failure and electrical dyssynchrony, particularly in those with reduced left ventricular (LV) ejection fraction and wide QRS complexes due to left bundle branch block.^1,2^ CRT is commonly achieved by transvenous placement of an LV lead into a lateral vein of the coronary sinus (CS). Anatomical variations and venous anomalies can present significant technical challenges during the procedure. We present a case of a 65-year-old man with a history of aortic valve replacement and heart failure who underwent successful CRT-defibrillator (CRT-D) implantation. Despite anatomical challenges such as CS ostial stenosis, LV lead implantation succeeded via the vein of Marshall (VOM).

## Case presentation

A 65-year-old man with a history of aortic valve replacement 38 years ago and persistent heart failure was referred to our clinic for CRT implantation. He had New York Heart Association class II heart failure symptoms and a left bundle branch block with a QRS duration of 170 ms on surface electrocardiogram. A transthoracic echocardiography revealed an LV ejection fraction of 30% despite optimal medical therapy.

CRT implantation was performed under local anesthesia. Following dissection of a subclavian pocket within the pectoral muscle, the right ventricular (RV) lead was positioned at the apical–septal region of the RV. A CS cannulation sheath was then introduced into the right atrium (RA), and CS entry was attempted. Although the CS ostium could be engaged, we were unable to advance any catheter further. Multiple attempts using a Marinr ablation catheter (Medtronic, Minneapolis, MN, USA) and an Amplatz left catheter (DxTerity™; Medtronic) were unsuccessful.

**Figure 1:**
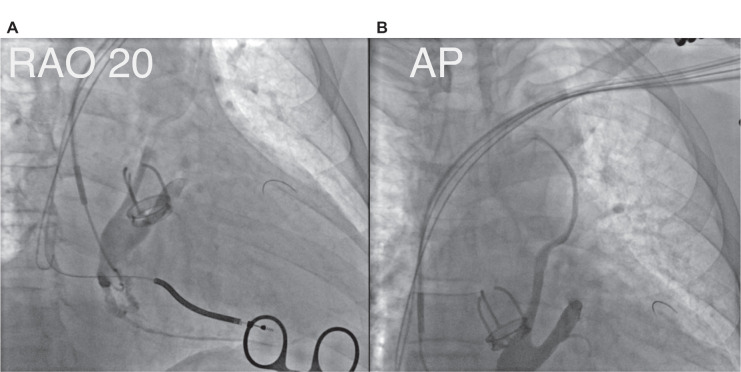
**A:** Atresia of the coronary sinus ostium (right anterior oblique view, 20°). **B:** Vein of Marshall (anteroposterior view).

Subsequently, contrast injection at the CS ostium revealed an upward-directed vein originating from the CS and draining into the subclavian vein **(Figure 1)**. This finding raised suspicion for the presence of a VOM. Attempts to cannulate the VOM using a Judkins right catheter (Medtronic) from the puncture site were unsuccessful. Due to retrograde blood flow, adequate opacification of the vein with contrast was limited.

**Figure 2:**
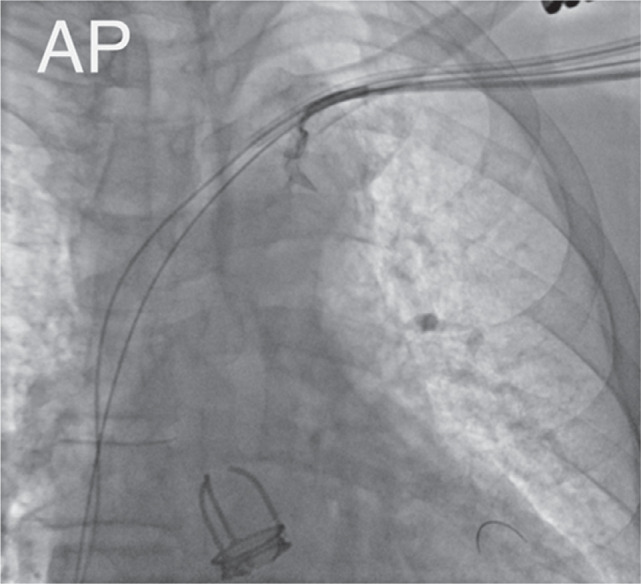
Cannulation of the vein of Marshall (anteroposterior view).

**Figure 3:**
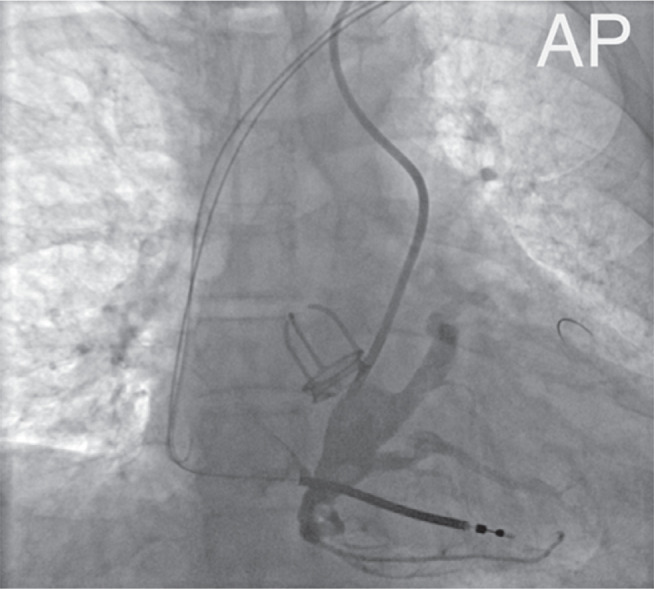
Visualization of the coronary sinus ostium and lateral vein (anteroposterior view).

**Figure 4:**
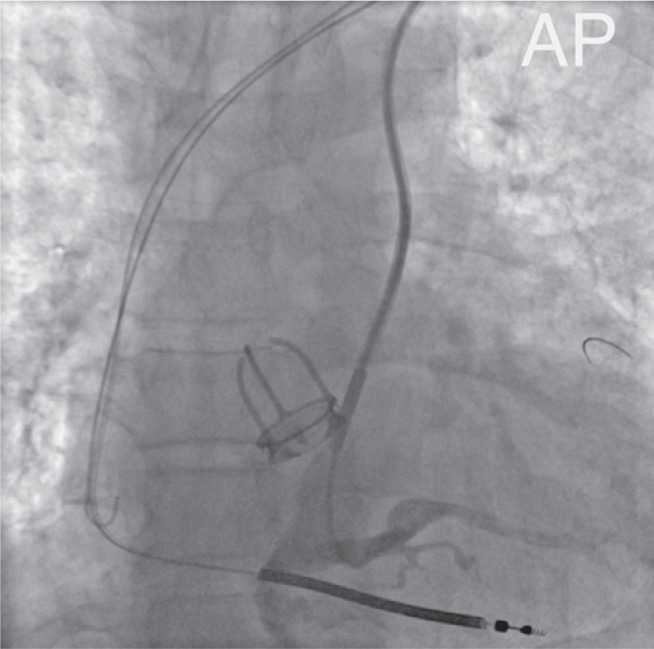
Cannulation of the lateral vein (anteroposterior view).

**Figure 5:**
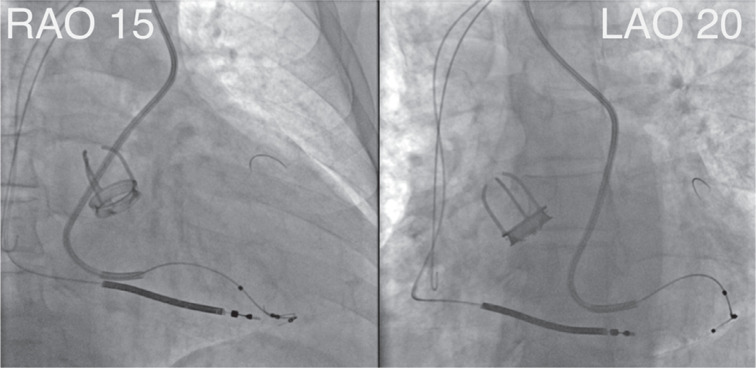
Final right (15°) and left (20°) anterior oblique views of the left ventricular lead position.

After exchanging the catheter for a Cobra2 5Fr (Radifocus™ Optitorque™; Terumo, Tokyo, Japan), the distal portion of the vein was successfully engaged **(Figure 2)**. A hydrophilic guidewire was then advanced into the lateral vein. The CS cannulation sheath was subsequently introduced into the CS body over the Cobra2 catheter. The contrast injection confirmed the course of the lateral vein and revealed occlusion at the level of the CS ostium **(Figure 3)**. The lateral vein was engaged using a Cobra2 catheter, and a 0.014″ coronary guidewire was advanced into this target vein **(Figure 4)**. Over the guidewire, a quadripolar LV lead (Attain Performa MRI SureScan™; Medtronic) was then advanced deep into the lateral vein with adequate lead stability and satisfactory pacing and sensing parameters, without any diaphragmatic stimulation **(Figure 5)**. The RA lead was implanted via left axillary venous access.

Written informed consent was obtained from the patient.

## Discussion

CRT is a cornerstone in the treatment of patients with heart failure and wide QRS complexes due to left bundle branch block. However, despite advancements in lead implantation techniques, some patients are unable to benefit from CRT due to technical failures during the procedure. According to a meta-analysis by Gamble et al., the failure rate for left LV lead placement ranges from 2.4%–5.4% during CRT device implantation.^3^ One of the causes of implantation failure is the inability to cannulate the CS ostium due to anatomical variations. CS ostium atresia is a rare congenital anomaly, with only a limited number of reported cases in the literature.^4^ It is frequently associated with a persistent left superior vena cava (LSVC). In such cases, venous drainage from the CS typically flows retrogradely through the LSVC into the innominate vein.^5^ This explains why, in our case, LSVC was not visualized during contrast imaging of the subclavian vein prior to venous puncture. This anomaly is usually benign and asymptomatic, often going unnoticed until procedures involving the CS are attempted or during advanced imaging. For this reason, many cases have historically been identified postmortem.

CS ostium atresia presents a significant anatomical challenge for LV lead implantation. In such cases, the LSVC or VOM can provide an alternative route for lead placement. However, this approach requires considerable operator experience and the availability of specialized tools due to the acute angulation and small caliber of the venous pathway.

The anatomy of the CS and its target branches, as well as their suitability for lead implantation, can be assessed via coronary angiography with levophase imaging. Unfortunately, in our patient, this was not performed, and the occlusion of the CS ostium became apparent only during the procedure.

Implantation of an LV lead via the VOM is technically challenging for several reasons. First, retrograde blood flow within the VOM often limits adequate contrast opacification, making visualization of the vein difficult. Second, the acute angulation and narrow connection between the VOM and the CS body pose significant challenges for catheter and guidewire navigation. In our case, multiple attempts to cannulate the VOM and access the lateral vein were made using a Judkins right diagnostic catheter and a Medtronic Marinr ablation catheter, which were unsuccessful. Successful cannulation was achieved only after switching to a Cobra2 catheter, which allowed engagement of the distal portion of the vein. Furthermore, a hydrophilic guidewire was essential to navigate the tortuous venous anatomy and reach the target lateral vein.

A literature search revealed only a limited number of published cases describing LV lead implantation via the VOM in the presence of CS ostial atresia.^6,7^ In most of these cases, successful lead placement was facilitated by the presence of a persistent LSVC, which was absent in our patient.

What distinguishes our case is that LV lead implantation was achieved only after multiple failed attempts using conventional catheters. Success was obtained using a Cobra2 catheter in combination with a hydrophilic guidewire, which enabled engagement of the distal VOM and access to the target lateral vein.

The procedure can be seen in **Video 1**.

## Conclusion

CS ostium atresia is a rare but significant anatomical variation that presents unique challenges during CRT implantation. Awareness of this condition, coupled with advanced imaging techniques and a flexible approach to the procedure, can improve success rates in these complex cases.

## Supporting information

Video 1: Procedural video.

## References

[1] Bristow MR, Saxon LA, Boehmer J (2004). Cardiac-resynchronization therapy with or without an implantable defibrillator in advanced chronic heart failure. N Engl J Med.

[2] Cleland JG, Abraham WT, Linde C (2013). An individual patient meta-analysis of five randomized trials assessing the effects of cardiac resynchronization therapy on morbidity and mortality in patients with symptomatic heart failure. Eur Heart J.

[3] Gamble JHP, Herring N, Ginks M, Rajappan K, Bashir Y, Betts TR (2016). Procedural success of left ventricular lead placement for cardiac resynchronization therapy: a meta-analysis. JACC Clin Electrophysiol.

[4] Santoscoy R, Walters HL, Ross RD, Lyons JM, Hakimi M (1996). Coronary sinus ostial atresia with persistent left superior vena cava. Ann Thorac Surg.

[5] Jha NK, Gogna A, Tan TH, Wong KY, Shankar S (2003). Atresia of coronary sinus ostium with retrograde drainage via persistent left superior vena cava. Ann Thorac Surg.

[6] Nath RK, Singh AP, Kuber D, Kayal V (2022). Marshall to the rescue in cardiac resynchronization therapy: Left ventricular lead placement in coronary sinus ostial atresia. Indian Pacing Electrophysiol J.

[7] Bajwa A, Dhoot J, Gupta S (2021). Coronary sinus lead placement in patients with coronary sinus ostial atresia: an innovative approach. JACC Case Rep.

